# On the Immediate Cure of Piles

**Published:** 1877-04

**Authors:** 


ON THE IMMEDIATE CURE OF PILES.—Mr. Reeves, of Edinburgh, has adopted a plan of treating internal piles to which he has given the term "immediate cure." The operation is rapid and the entire treatment short, as compared with the ordinary method, viz., by nitric acid, ligature, clamp and cautery. He thinks, moreover, that it is free from danger and does not always require an anæsthetic. The piles being well down, are punctured to their bases by the conical tip of the gas cautery (Dr Paquelin's). The number of the punctures varies with the number and size of the piles, a pile the size of a half walnut requiring two or three. A dull red heat should be employed, and the point of the instrument is to be gently rotated while it is within; otherwise a portion of the eschar will be withdrawn, and then hemorrhage may ensue. Ulcers or fissures should be cauterized at the same time. Should there be any oozing, a touch of the cautery will stop it. The piles are then to be returned and a half-grain morphia suppository inserted. After the bowels have been confined for four or five days, a warm injection is to be given, and followed upon the succeeding day by a laxative. At the expiration of a week the patients are discharged. Of eighteen cases thus operated on, two were not allowed out for ten days, and one for a fortnight, but in these cases there was some uterine or urinary complication. All the patients were examined subsequently, and it was exceedingly difficult to discover by the finger or the speculum that there were any cicatrices following the operation.—*Lancet.*

